# CyberKnife robotic spinal radiosurgery in prone position: dosimetric advantage due to posterior radiation access?

**DOI:** 10.1120/jacmp.v15i4.4427

**Published:** 2014-07-08

**Authors:** Christoph Fürweger, Christian Drexler, Alexander Muacevic, Berndt Wowra, Erik C. de Klerck, Mischa S. Hoogeman

**Affiliations:** ^1^ European CyberKnife Center Munich Munich Germany; ^2^ Erasmus MC – Cancer Institute Rotterdam The Netherlands

**Keywords:** spinal radiosurgery, prone position, image guidance, CyberKnife

## Abstract

CyberKnife spinal radiosurgery suffers from a lack of posterior beams due to workspace limitations. This is remedied by a newly available tracking modality for fiducial‐free, respiration‐compensated spine tracking in prone patient position. We analyzed the potential dosimetric benefit in a planning study. Fourteen exemplary cases were compared in three scenarios: supine (PTV=CTV), prone (PTV=CTV), and prone position with an additional margin (PTV=CTV+2 mm), to incorporate reduced accuracy of respiration‐compensated tracking. Target and spinal cord constraints were chosen according to RTOG 0631 protocol for spinal metastases. Plan quality was scored based on four predefined parameters: dose to cord ($D_{0.1\text{cc}}$ and $D_{1\text{cc}}$), high dose ($V_{10\text{Gy}}$), and low dose ($V_{4\text{Gy}}$) volume of healthy tissue. Prescription dose was 16 Gy to the highest isodose line encompassing 90% of the target. Results were related to target size and position. All plans fulfilled RTOG 0631 constraints for coverage and dose to cord. When no additional margin was applied, a majority of eight cases benefitted from prone position, mainly due to a reduction of $V_{4\text{Gy}}$ by 23% ± 26%. In the 2 mm prone scenario, the benefit was nullified by an average increase of $V_{10\text{Gy}}$ by 43% ± 24%, and an increase of $D_{1\text{cc}}$ to cord (four cases). Spinal cord $D_{0.1\text{cc}}$ was unchanged (<±1Gy) in all but two cases for both prone scenarios. Conformity (nCI) and number of beams were equivalent in all scenarios, but supine plans used a significantly higher number of monitor units (+16%) than prone. Posterior beam access can reduce dose to healthy tissue in CyberKnife spinal radiosurgery when no additional margin is applied. When a target margin of 2 mm is added, this potential gain is lost. Relative anterior‐posterior position and size of the target are selection criteria for prone treatment.

PACS numbers: 87,53.Ly, 87.55.D‐

## I. INTRODUCTION

Spinal radiosurgery is an increasingly utilized technique,^(1)^ where ablative radiation doses are delivered to spinal lesions in a highly accurate and conformal manner. Current radiosurgery systems apply noninvasive image‐guided tracking of the skeletal spine structure for precise targeting of the lesion.^2^, ^3^

The CyberKnife (CK) (Accuray Inc., Sunnyvale, CA) has recently been described in a comprehensive review.^(4)^ Briefly, it is comprised of a compact 6 MV linear accelerator mounted on a robotic arm that is guided by a stereoscopic kV imaging system for target visualization and a 3D camera array for continuous monitoring of respiratory motion. Different software applications allow for tracking of stationary and moving targets. The CK delivers nonisocentric and noncoplanar circular beams from a large solid angle around the patient.

In a noncoplanar setting, the available beam direction space has been shown to affect plan quality.^(5)^ Due to the specific geometry of the CK layout, the space below the treatment table plane cannot be accessed by the linac. This prohibits posterior radiation beams for patients in supine position. Until recently, the CK fiducial‐free spinal image‐guidance modality “Xsight skeletal structure tracking” (Accuray Inc.)^(6)^ was limited to supine patient position because respiratory motion of spinal targets in prone position could not be adequately managed. As a consequence of supine position and CK workspace limitations, beams aimed at very posterior spinal lesions had to traverse a substantial length of healthy tissue on their way to the target.

With the recent release of CK software version 9.6 in 2012, a new tracking modality called “Xsight Spine Prone” (Accuray Inc.) combines the current spine image registration algorithm^(7)^ with dynamic compensation of respiratory motion. Thus, spinal targets can now also be targeted in prone position. However, while supine position tracking of the static spine is known to achieve submillimeter precision in the clinical situation,^8^, ^9^ a targeting error of up to 2 mm has been determined for dynamic tracking of lung lesions moving with respiration.^(10)^

The treating physician is now expected to pick one of two different fiducial‐free options to treat a particular spinal lesion — highly accurate treatment in supine position at the expense of a potentially greater effective depth of the target, or dynamic tracking in prone position with the benefit of posterior radiation access, but sacrificing accuracy of delivery. Our objective was to quantitatively assess the dosimetric potential of both options in a comparative planning study. We created treatment plans in supine and prone orientation for 14 exemplary spinal lesions. To incorporate the aspect of differing accuracy, we compared a 0 mm and a 2 mm margin scenario for the prone target to a 0 mm margin supine reference, and quantified the potential benefit of posterior beam access in dependence of clinical case characteristics. The data were used to identify objective criteria for selecting the appropriate CK spinal tracking modality.

## II. MATERIALS AND METHODS

### A. Case characteristics and contouring

Fourteen cases were selected from a library of more than 400 CyberKnife spinal cases. Main criterion was principle suitability for prone treatment with respect to location of the lesion (i.e., thoracic (T9 or lower), lumbar, and sacral spine). Additionally, cases were chosen to include patients of different size and target configurations. Cases with metal implants in the target area were excluded to minimize dosimetric uncertainty. For all cases, a planning CT scan with 1 to 1.5 mm slice spacing and the clinical target volume (CTV) used for treatment was available, with the CT dataset exceeding the target by at least 10 cm in superior and inferior direction. The maximum size of the CTV was restricted to two vertebral bodies (mean 43.8 cm^3^, median 45.9 cm^3^, range 5.9 to 85.7 cm^3^) in accordance with eligibility for single‐fraction approach.

#### A.1 Supine plans

The CTV was used as target volume with no additional margin. The main organ at risk (OAR), the spinal cord, was contoured over the whole length of the simulation CT scan. In order to accurately assess the high‐risk area of the cord close to the target, an additional subvolume was created, in accordance with the ongoing RTOG 0631 SBRT study for localized spine metastases:^(11)^ the “partial spinal cord volume” was defined as the part of the spinal cord that extends from 5–6 mm superior to 5–6 mm inferior of the target.

#### A.2 Prone plans

The planning CT scan and contour sets were flipped by 180° along the roll axis and used as a basis for prone plans. Two margin scenarios were assessed: i) 0 mm margin to directly analyze the impact of posterior radiation access, and ii) an expanded planning target volume (PTV) given by the CTV+2 mm isotropic margin in order to incorporate the reduced accuracy in delivery. This value is derived from CK tracking data of moving tumors^(10)^ with an excursion of up to 2 cm, which is a reasonably conservative approach because spine motion in prone position has so far been reported to be in the millimeter range.^8^, ^12^, ^13^ In comparison, we used a zero‐margin concept in the supine scenario, which is motivated by the submillimeter precision reported for a series of 260 spine treatments with CK.^(9)^ The expansion by 2 mm corresponds to an increase of the target volume by 37.4%±8.4% (min 26.7%, max 61.0%).

### B. Planning technique and objectives

The CK MultiPlan (Accuray Inc.) v3.5 treatment planning software with sequential optimization algorithm^(14)^ was used for inverse plan optimization. This algorithm identifies a suitable subset from a total of more than 2000 randomly generated candidate beams. Beam diameters are selected by the user from 12 circular aperture sizes (5 to 60 mm at 80 cm distance). User‐defined dose limits, planning goals, and beam parameters are translated into separate objective functions that are solved in a hierarchical manner.

Inverse planning goals can be intrinsically contradicting (e.g., target coverage vs. dose to an adjacent OAR). In order to generate meaningful, comparable data, all scenarios of interest must adhere to the same, predefined planning objectives. We have defined two primary and several secondary objectives. Primary objectives must be achieved with highest priority and are not compromised during optimization for secondary goals. The primary objectives include target and cord dose, following the RTOG 0631 protocol for spine metastases.^(11)^ The first objective is to cover at least 90% of the target with the 16 Gy isodose line (IDL). The second objective is to limit the dose to the spinal cord, so that $V_{14\text{Gy}}$ and $V_{10\text{Gy}}$ of the partial cord volume do not exceed 0.035 and 0.35 cm^3^, respectively.

Secondary goals were minimization of the high‐dose volume of normal tissue $\left(V_{10\text{Gy}}\right)$, further reduction of dose to the cord $\left(D_{1\text{cc}},D_{0.1\text{cc}}\right)$, and decrease of the global low‐dose volume (”dose bath”, $V_{4\text{Gy}}$). Prioritization of secondary goals was decided by the user with regard to the specific case configuration (e.g., if the dose to cord is inherently low due to above‐average distance of target to cord, emphasis was put on reduction of $V_{10\text{Gy}}$ and $V_{4\text{Gy}}$, and vice versa).

Dose prescription was 16 Gy to the highest IDL encompassing 90% of the target (CTV or 2 mm PTV). No effort was made to specifically adjust dose homogeneity within the target because very different isodose levels (50% to 85%) are being used clinically.^15^, ^16^

An upper limit of 30000 linac monitor units (MUs) and 300 beams was defined to allow for delivery within a clinically reasonable timeframe of up to 90 mins. Plans that did not meet this criterion were discarded and reoptimized.

### C. Plan quality metrics and case classification

Four main metrics for plan quality were chosen, in correspondence to secondary optimization goals:

- spinal cord $D_{0.1\text{cc}}$, corresponding to the maximum dose area of the main OAR
- spinal cord $D_{1\text{cc}}$, as a general indicator for cord toxicity
- total $V_{10\text{Gy}}$ subtracted by the CTV (i.e., $V_{10\text{Gy}}$ outside of the CTV), as a measure for the high‐dose normal tissue volume
- total $V_{4\text{Gy}}$ subtracted by the CTV (i.e., $V_{4\text{Gy}}$ outside of the CTV), as a measure for the low‐dose normal tissue volume (”dose bath”) to the patient

For further plan characterization, the following parameters were assessed, even though they were not defined as optimization goals: the number of MUs and beams; mean effective dosimetric depth (by averaging the electron density‐corrected depth of all beams from patient entrance point to the reference point inside the tumor) as an indicator for beam access and MU efficiency; and normalized conformity index (nCI) as given by:

(1) $$nCI=\frac{TV}{TV\text{Pr}}\times \frac{\text{Pr}V}{TV\text{Pr}}$$

where *TV* is the target volume, *PrV* is the total tissue volume that receives the prescription isodose or more, and *TVPr* is the target volume that receives the prescription isodose or more.

Anterior‐posterior (AP) target location was recorded twofold: 1) target to surface distance (in cm, from the target center to the anterior or posterior patient surface); 2) relative AP target position (distance in cm in AP direction, from the target center to the AP patient center point, with negative values for a target offset in anterior and positive values for an offset in posterior direction). Observed differences in plan quality metrics, nCIs, and number of MUs and beams were tested for statistical significance (null hypothesis) using paired Student's *t*‐tests.

In order to identify cases with an overall benefit due to prone position treatment, (potentially conflicting) results from different quality metrics need to be merged. We have adopted a simple classification scheme to summarize the four main quality metrics and rate all 14 cases on a discrete scale from −4 to +4, with negative values corresponding to an advantage for supine position and positive values for prone. Detailed classification criteria and thresholds are given in Table 1.

**Table 1:** Classification criteria with supine plans used as reference. Prone plans within the indicated range of the supine reference were scored as no change. Improvements (reduction of indicated parameters) were scored as +1, degradation as −1. The range of results was −4;+4

*Parameter*	*Cord* $D_{0.1cc}$	*Cord* $D_{1cc}$	*Total* $V_{10Gy}-CTV$	*Total* $V_{4Gy}-CTV$
Range	±1Gy	±1Gy	±10%	±10%

## III. RESULTS

### A. Primary objectives

Target coverage of more than 90% with the 16 Gy IDL was achieved for all cases. Mean target coverage was 91.6%±0.7%,91.2%±0.8%, and 91.4%±0.9% for supine, prone 0 mm and prone 2 mm margin scenarios. Spinal cord dose limits $\left(V_{14\text{Gy}}\leq 0.035\;{\text{cm}}^{3},V_{10\text{Gy}}\leq 0.35\;{\text{cm}}^{3}\right)$ were not exceeded. Spinal cord maximum dose was below 14 Gy in 40 of 42 plans, with $V_{14\text{Gy}}<0.005\;{\text{cm}}^{3}$ for the remaining. Spinal cord $V_{10\text{Gy}}$ was $0.08\pm 0.12\;(\max0.31)\;{\text{cm}}^{3},0.06\pm 0.10\;(\max0.29)\;{\text{cm}}^{3}$, and $0.10\pm 0.14\;(\max0.35)\;{\text{cm}}^{3}$ for supine, prone 0 mm, and prone 2 mm plans. For all plans, spinal cord high‐dose volume was limited to the cord surface, with no high‐dose streaks traversing the cord.

### B. Secondary goals and quality metrics

For plan quality metrics, supine plans are considered as the standard. Prone plan results are presented as an increase or decrease compared to the corresponding supine reference, unless stated otherwise.

**Figure 1:**
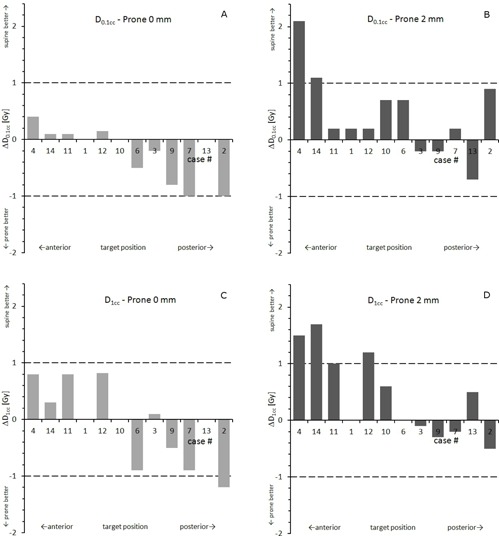
Difference in dose to spinal cord with respect to the supine reference plan given for prone 0 mm ((a): $D_{0.1\text{cc}}$; (c): $D_{1\text{cc}}$) and prone 2 mm ((b): D0.1cc; (d) D1cc) scenarios. Negative numbers indicate a decrease, positive an increase, in cord dose due to prone position. Cases are sorted according to target location (A→P).

Results for spinal cord $D_{0.1\text{cc}}$ and $D_{1\text{cc}}$ are shown in Figs. 1(a) to 1(d). The two sacral cases (Table 1, cases 5 and 8) were excluded because of negligible cord doses due to the long distance to the target. No significant difference could be ascertained for $D_{0.1\text{cc}}$ in the prone 0 mm scenario; average $D_{0.1\text{cc}}$ changed by −0.23±0.48 Gy (p=0.12), with two of the remaining 12 cases showing a substantial reduction of ≥1 Gy (Fig. 1(a)). Conversely, $D_{0.1\text{cc}}$ was increased by 0.43±0.73 Gy (p=0.06) for prone 2 mm plans, with an increase ≥1 Gy in two cases (Fig. 1(b)). Correspondingly, no significant difference could be observed for $D_{1\text{cc}}$ in both prone scenarios. $D_{1\text{cc}}$ changed by −0.06±0.70 Gy (p=0.78) for prone 0 mm plans, with a reduction ≥1 Gy in one case (Fig. 1(c)), but increased by 0.45±0.75 Gy (p=0.06) for prone 2 mm plans, with an increase ≥1 Gy in four cases (Fig. 1(d)).

**Figure 2:**
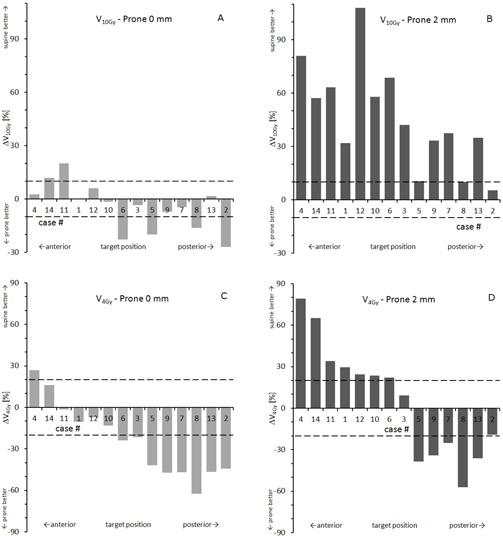
Volume of healthy tissue subjected to 10 Gy and 4 Gy given as a percentage increase/decrease with respect to the supine reference plan ((a), (b): prone 0 mm; (c), (d): prone 2 mm). Negative numbers indicate a decrease, positive an increase in volume due to prone position. Cases are sorted according to target location (A→P).

Normal tissue receiving more than 10 Gy (or 4 Gy) was defined as the total $V_{10\text{Gy}}$ (or $V_{4\text{Gy}}$) minus CTV. Figures 2(a) to 2(d) show the prone data as a percent increase or decrease. For $V_{10\text{Gy}}$ in prone 0 mm plans, no significant difference was observed in comparison to the supine reference, with an average change of −4.4%±13.4% (p=0.45). Four cases exhibited a decrease; two cases, an increase of more than 10% (Fig. 2(a)). The PTV expansion in the prone 2 mm scenario resulted in a huge increase of $V_{10\text{Gy}}$ by 41.0%±23.8% (p=0.02), which was larger than 10% in 12 of 14 cases (Fig. 2(b)).

The healthy tissue volume receiving a low dose as expressed by $V_{4\text{Gy}}$ strongly depended on the anterior/posterior target position (Figs. 2(c) and 2(d)). $V_{4\text{Gy}}$ was significantly reduced for prone 0 mm by 23.2%±26.4% (p=0.02), with a decrease of ≥20% in eight cases and an increase of  in one case (Fig. 2(c)). For prone 2 mm plans, no significant difference was found for $V_{4\text{Gy}}$, with an average change of +5.5%±41.2% (p=0.73). A group of seven cases exhibited an increase, five a decrease of ≥20% (Fig. 2(d)).

**Figure 3:**
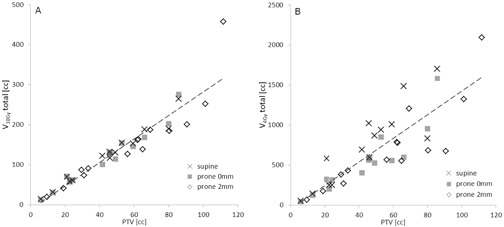
Total tissue volume subjected to 10 Gy (a) and 4 Gy (b) as a function of target size.

To illustrate the different outcome for 10 Gy and 4 Gy dose levels, total $V_{10\text{Gy}}$ and $V_{4\text{Gy}}$ (including the target) were plotted against target volume (Fig. 3). We found a pronounced linear relationship between target volume (PTV) and $V_{10\text{Gy}}$ for all three scenarios (Fig. 3(a)), with little spread of the data points. In comparison, while $V_{4\text{Gy}}$ is also dependent on target volume, data points show a much larger spread (Fig. 3(b)) than $V_{10\text{Gy}}$, reflecting the strong dependence of this metric on A/P target position (Figs. 2(c) and 2(d)).

**Figure 4:**
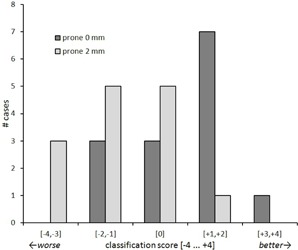
Improvement/degradation of plan quality due to prone position using classification criteria in Table 1. Cases are grouped to indicate major (+3,+4) and minor (+1,+2) improvement, no change (0), and minor (−1,−2) and major (−3,−4) degradation.

Classifying the cases according to the criteria defined in Table 1, a majority of eight cases benefited from prone patient position when no additional margin was applied, three showed no change, and three cases had an inferior dose distribution (Fig. 4). With an added PTV margin of 2 mm, the outcome is reversed: prone 2 mm plans were worse for eight cases, five cases showed no change, and only one showed improvement compared to supine.

### C. Additional plan characteristics

**Figure 5:**
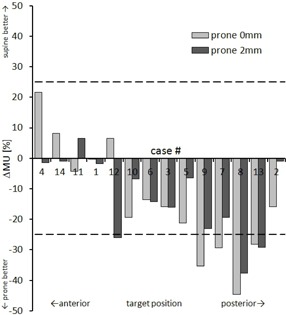
Percentage increase/decrease in monitor units with respect to the supine reference plan. Cases are sorted according to target location (A→P).

Further plan characteristics that had not been defined as optimization goals were assessed: differences in conformity were insignificant (p=0.16), with average nCIs of 1.25±0.11 and 1.23±0.09, for supine and prone 0 mm plans. All plans stayed below the predefined 300 beams and 30k MU limits. No significant difference in the number of beams was observed (p≥0.4), with 236±30,238±42, and 236±30 for supine, prone 0 mm, and prone 2 mm plans. The number of MUs was similar for prone 0 mm plans $\left(18.7\times 10^{3}\pm 4.6\times 10^{3}\right)$ and prone 2 mm plans $\left(19.0\times 10^{3}\pm 4.0\times 10^{3},p=0.74\right)$. Significantly more MUs were used in the supine scenario $\left(21.9\times 10^{3}\pm 3.9\times 10^{3},+16\%,p\leq 0.01\right)$. Prone position reduced the number of MUs by more than 25% in four (0 mm margin) / three (2 mm margin) cases (Fig. 5).

**Figure 6:**
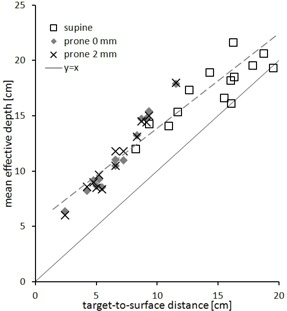
Mean effective depth to the target as a function of target‐to‐surface distance along the A/P axis. The anterior surface is used for supine plans, the posterior for prone.

**Figure 7:**
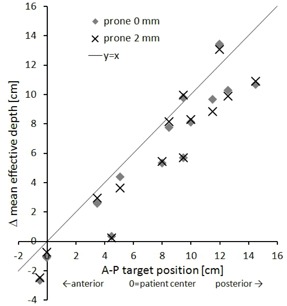
Change in mean effective depth as a function of relative AP target position, with $\Delta d_{\text{eff}}=d_{\text{eff}\_\text{prone}}-d_{\text{eff}\_\text{supine}}$. AP target position is given as cm offset from the patient AP center, with negative numbers indicating anterior position and positive for posterior.

In order to analyze beam access and use of the CyberKnife workspace in dependence of target location, we plotted mean effective depth as a function of target‐to‐surface distance, with the anterior/posterior skin as boundary for supine/prone plans (Fig. 6). Mean effective depth correlated positively with target‐to‐surface distance. For superficial targets, mean effective depth was more than twice the target‐to‐surface distance. Accordingly, the difference in effective depth between supine and prone plans correlated with the relative AP target position (Fig. 7).

## IV. DISCUSSION

Literature on prone treatments and relevance of posterior beam access with CyberKnife is very scarce. In a recent feasibility study on fiducial‐free treatment of spine patients in prone position treatments with CK,^(16)^ a potential dosimetric advantage has been suggested, based on standardized targets in only one patient case. We were able to demonstrate that in an exemplary group of 14 patients, a majority of spinal targets treated with CyberKnife could benefit from posterior radiation access. However, this finding needs to be put into perspective for two reasons. First, the potential advantage is small and mainly refers to a reduction of the low‐dose volume in the periphery (Fig. 2). Second, this result is based on the assumption of equal accuracy of static delivery in supine position and dynamic tracking in prone position.

At first glance, the lack of posterior beams occurs to be a significant drawback for CyberKnife spinal radiosurgery. Therefore, the relatively small gain in plan quality due to prone patient position might seem unexpected. However, this result can be explained by the CK node distribution and usage. With CyberKnife, high conformality and a steep dose falloff around the target are achieved by isotropic dose delivery. Thus, the contribution of lateral and oblique beams is crucial for plan quality. These beams use a longer path to the target, and share similar dosimetric characteristics for prone and supine plans. Hence, the mean effective depth is significantly larger than the AP target‐to‐surface distance, especially for superficial targets (Fig. 6). Essentially, the relative AP target position is only important for a subset of nodes located in a limited solid angle directly above table. As a consequence, the difference between prone and supine plans, in terms of mean effective depth, is generally smaller than would be expected due to the relative AP target position (Fig. 7) and, therefore, the supposed dosimetric advantage of direct posterior access is diminished. This effect is amplified when an adjacent OAR is located posterior of the target (e.g., vertebral body metastasis, spinal cord). For this configuration, the optimizing system preferentially uses the sharp, lateral penumbra to create steep dose gradients, thus further promoting more lateral beam angles.

In our study, patients were rotated virtually rather than rescanned in prone position. Since this is an approximation of the actual patient geometry in prone position, we have deliberately selected generalized plan quality measures such as total high‐ $\left(V_{10\text{Gy}}\right)$ and low‐dose volumes $\left(V_{4\text{Gy}}\right)$, rather than reporting on doses to specific soft tissue OARs, in order to avoid the impact of patient deformation.

It has recently been reported that even in the lower lumbar spine, respiration induced target motion in prone position could not be cancelled out completely,^(13)^ arguing for the need for compensation of respiratory motion during delivery. However, dynamic tracking is known to be slightly less accurate than tracking of static targets.^10^, ^17^ So, when prone and supine plans are compared using identical target margins, differing accuracy translates into an overly optimistic evaluation of the prone scenario. We included this aspect by reassessing prone plans with an added margin of 2 mm. Since PTV size is a major determinant for high‐dose volume to healthy tissue (Fig. 3(a)), the dosimetric benefit of posterior access is challenged by the need to cover a larger target. In contrast, while the low‐dose volume also increases with PTV size to some extent (Fig. 3(b)), this can be balanced by advantageous target position (Fig. 2(d)). However, considering both aspects, our data show that in most cases, enlarging the target outweighs advantageous beam entry.

Of relevance for clinical decision making, we could identify favorable candidates for prone position treatment by classification of all cases (Fig. 4). There were only a few candidates where prone position treatment would still be a reasonable approach despite the 2 mm expansion. The corresponding targets shared two characteristics: very posterior position (3:1 anterior vs. posterior target‐to‐surface distance) and, with one exception, large volume $\left(\text{CTV}>40\;{\text{cm}}^{3}\right)$. When these two criteria are met, the healthy tissue volume receiving high doses will still increase by a relatively small amount, but a large decrease of the volume subjected to lower doses can be expected, which is coupled with a decrease in the number of required MUs.

## V. CONCLUSIONS

In CyberKnife spinal radiosurgery, dose to healthy tissue can potentially be reduced by allowing for posterior radiation access by placing the patient in prone position. However, when an additional target margin of 2 mm is added in order to compensate for lower accuracy in prone delivery, this advantage is lost in most cases. Size and the relative anterior‐posterior position of the target are the determining criteria to identify the few patients suitable for prone position treatment.

Overall, the benefit of posterior radiation access to spinal targets is small, at best. However, taking into account that every plan included in this study has met the RTOG 0631 requirements for target coverage and dose to the cord, the presented data also allow for a different interpretation: The lack of posterior beams in CyberKnife spinal radiosurgery is an insignificant issue.

## ACKNOWLEDGMENTS

The authors would like to thank Petr Jordan (Accuray Inc.) for conversion and adaptation of DICOM structures and datasets.

## Supporting information

Supplementary Material
