# Supplementary figures and images for: CyberKnife robotic spinal radiosurgery in prone position: dosimetric advantage due to posterior radiation access?
Source: J Appl Clin Med Phys. 2014 Jul 8;15(4):11–21. doi: 10.1120/jacmp.v15i4.4427 (PMC5875502; doi:10.1120/jacmp.v15i4.4427)

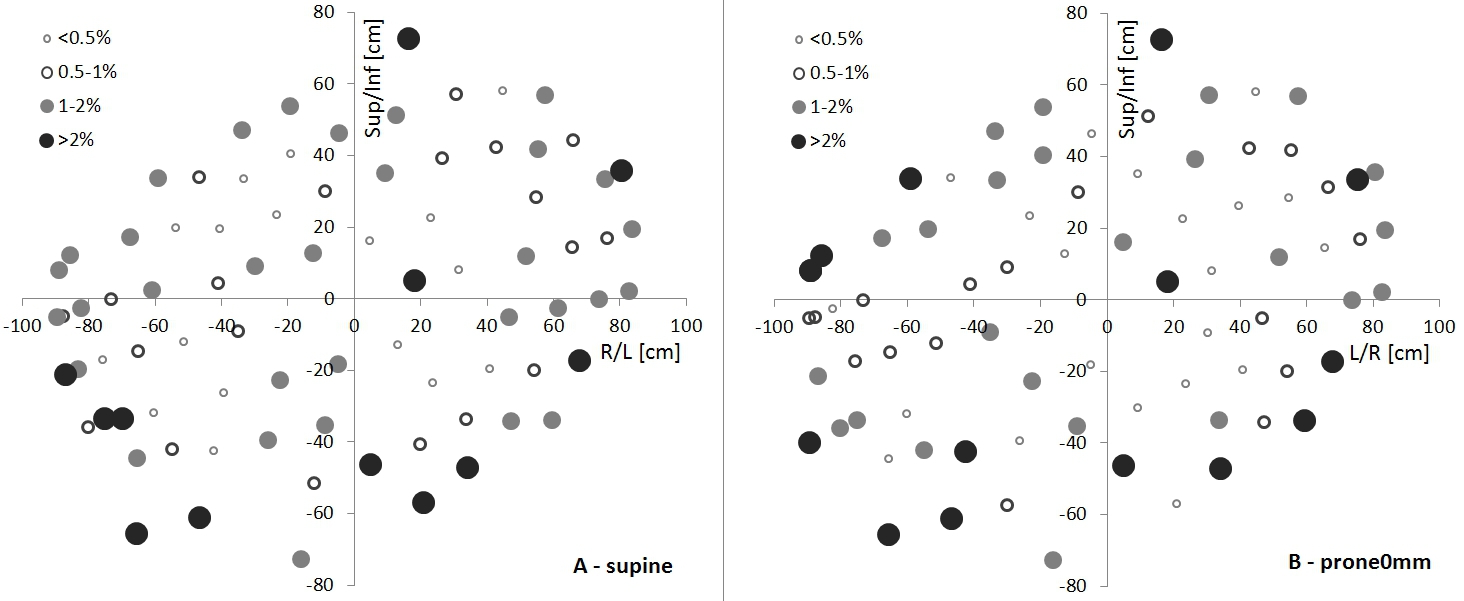

Supplement: Supplementary file 1 — Supplementary Material [file ACM2-15-11-s001.jpg]
